# MassSeg-Framework: A Breast Mass Detection and Segmentation Framework Based on Deep Learning and an Active Contour Model

**DOI:** 10.3390/life16040653

**Published:** 2026-04-12

**Authors:** Camila Zambrano, Noel Pérez-Pérez, Miguel Coimbra, Maria Baldeon-Calisto, Ricardo Flores-Moyano, José Ramón Mora, Oscar Camacho, Diego Benítez

**Affiliations:** 1 Colegio de Ciencias e Ingenierías “El Politécnico”, Universidad San Francisco de Quito USFQ, Quito 170157, Ecuador; czambrano@alumni.usfq.edu.ec (C.Z.); mbaldeonc@usfq.edu.ec (M.B.-C.); rflores@usfq.edu.ec (R.F.-M.); ocamacho@usfq.edu.ec (O.C.); dbenitez@usfq.edu.ec (D.B.); 2 INESC TEC, Faculdade de Ciências da Universidade do Porto, 4169-007 Porto, Portugal; mcoimbra@fc.up.pt; 3 Porter B. Byrum School of Business, Wingate University, Wingate, NC 28174, USA; 4 Departamento de Ingeniería Química, Instituto de Simulación Computacional (ISC-USFQ), Universidad San Francisco de Quito USFQ, Quito 170157, Ecuador; jrmora@usfq.edu.ec

**Keywords:** breast cancer, breast mass detection, breast mass segmentation, deep learning, active contour model

## Abstract

This work introduces the *MassSeg-Framework*, a fully automatic two-stage pipeline for breast mass analysis in mammography that integrates YOLOv11-based detection with Chan–Vese ACM refinement to achieve accurate mass localization and segmentation with a lightweight computational footprint. The framework was trained and evaluated on two publicly available datasets using consistent experimental protocols. In the detection stage, YOLOv11-nano was the most effective architecture, with a confidence threshold of 0.4, achieving statistically significant mAP50 values of 0.862 and 0.709 on the *dINbreast* and *dCBIS* datasets, respectively. These results confirm that a moderate threshold preserves clinically relevant true-positive candidates, which is particularly important for screening-oriented settings where missed lesions are costly. In the segmentation stage, the proposed framework achieved mean DICE scores of 0.721 and 0.700 on the test sets of the same datasets, demonstrating consistent overlap with expert annotations. Compared with state-of-the-art approaches that commonly assume lesion-centered ROIs or rely on heavier backbones, the proposed pipeline addresses a more realistic scenario by performing automatic detection followed by segmentation while maintaining substantially lower computational requirements. This balance between performance and efficiency makes the *MassSeg-Framework* a promising tool for scalable mammography analysis, particularly in resource-constrained environments or high-throughput screening workflows that require rapid processing.

## 1. Introduction

Cancer is a difficult disease characterized by the disorderly growth and division of cells [1] that disrupts the normal cell renewal process, leading to the formation of tumors [2]. Malignant tumors plague nearby tissues and spread to other body parts [2], while benign tumors do not invade neighboring tissues and can be removed successfully [2]. Breast cancer is the most common type of cancer in women globally. It can manifest in women after puberty, and the risk significantly increases with age. In 2020, the World Health Organization (WHO) reported that 2.3 million women were diagnosed with breast cancer, and 685,000 died from this disease [3]. Thus, early detection is vital to minimizing mortality.

Standard breast lesion detection protocols in healthcare centers are well established. Experts commonly recommend and use primary techniques, such as regular breast self-examinations [4,5], clinical breast examinations [5], mammograms [6,7,8,9,10,11], double reading of mammograms [12], ultrasound imaging [8,11,13], magnetic resonance imaging (MRI) [8,9,11], and biopsies [10], with the latter being the most invasive method. Mammogram screening is one of the most effective modalities because it can detect malignant lesions at an early stage before symptoms appear. Microcalcifications and masses are two common breast cancer lesions [14]. The first are tiny bright spots in the image that are easy to detect and segment from the background. The second group are more complex lesions due to their similarity to surrounding tissues and associated factors, such as breast density and variations in lesion shape, texture, and location, which can lead to incorrect diagnoses [14,15,16]. Like with other imaging-based techniques, finding breast lesions through screening is challenging for radiologists, with between 10% and 30% of breast cancer cases missed, around 80% of patients sent for an extra revision with a normal outcome, and 40% of biopsies resulting in a benign diagnosis [17,18].

Artificial intelligence-based detection and classification tools offer valuable support to experts analyzing mammograms, demonstrating that they can detect 20% more cancer cases than the radiologist’s double-reading routine [19] and provide a second opinion on potential benign or malignant mass lesions. Shallow learning-based methods such as support vector machines (SVMs) [20], random forests (RFs) [21], k-Nearest Neighbors (kNNs) [22], decision trees (DTs) [23], and artificial neural networks (ANNs) [24] have been favored to solve the mass lesion detection and classification problem, but they require a feature vector database extracted from medical images, patient records, and other relevant data sources for the learning task [25]. Digital image processing techniques, such as segmentation models based on active contour models (ACMs), have also been explored and applied [26,27,28]. These models cannot detect breast lesions in images on their own. Thus, they are linked to either manual or automatic identification of the image’s region of interest (lesion area). However, once the region is identified, they help accurately separate breast lesions from the background, enabling radiologists and healthcare professionals to focus on potential malignancies and achieve more precise diagnoses [29].

With significant advances in medical imaging technologies, innovative approaches, such as deep learning methods that use raw image data, have become necessary. They involve training complex neural network architectures to automatically learn patterns and representations from vast, annotated mammogram datasets [25]. Deep neural networks (DNNs) [30,31,32,33,34] are the most useful since they are powerful image feature extractors built on You Only Look Once (YOLO) detectors [35,36,37], Unet segmentation models [38,39,40], and the RCNN (Region-Based Convolutional Neural Network) detector family [41,42,43], which are less popular in this area.

Most existing methods focus on detecting and classifying mass lesions, as these tasks are less complex than segmentation. However, according to the American College of Radiology (ACR), the breast lesion malignancy grade in the BI-RAD (Breast Imaging-Reporting and Data System) scale varies following the shape of the lesion [44]. Well-defined, rounded contours are always benign (BI-RAD 1 and 2). In contrast, ill-defined and irregular contours are malignant (BI-RAD 5 and 6). However, the decision limit between the last benign grading (BI-RAD 3) and the initial malignant grading (BI-RAD 4) is very difficult to determine, suggesting invasive biopsy-based procedures [44]. Other studies combined detection and segmentation tasks into a single stage, such as U-Net-based methods. Although these approaches can be very effective, their operation on the entire image and increased model complexity often lead to higher memory usage, longer run times, and greater hardware demands, which can limit scalability in high-throughput screening workflows or in environments with diverse computational resources.

To overcome these limitations, we propose *MassSeg-Framework*, an automatic two-step breast mass segmentation method that combines the state-of-the-art YOLOv11 architecture with a Chan–Vese ACM to detect and locally segment mass lesions on mammography images at a lower computational cost. The *MassSeg-Framework* leverages the strengths of YOLOv11-based detectors to find candidate regions of interest (ROIs) and the versatility of ACMs for mass segmentation within these ROIs. Unlike ROI-dependent segmentation approaches, the proposed framework explicitly addresses lesion localization and contour delineation in a unified automatic pipeline while avoiding the increasing computational load of full-image one-stage segmentation architectures. The main contributions related to the proposal are described below.

- Computationally efficient algorithm: since the YOLOv11 architecture is built to make single-shot multi-scale object detections, integrating it into the proposed *MassSeg-Framework* results in a lower algorithm complexity, lower memory usage, and a more scalable solution for detecting breast mass lesions in large databases than the family of region-based models, such as RCNN and the state-of-the-art Unet model, which usually require more computational resources.
- Local mass lesions segmentation at a lower cost: ACMs primarily rely on minimizing energy functions using the Mumford–Shah function and level sets, along with an iterative segmentation curve deformation process, resulting in local mass contour determination with reduced hardware requirements instead of training deep learning architectures for this purpose.

The rest of the paper is organized as follows: the Related Works section provides an overview of related approaches. The Materials and Methods section provides a detailed description of the databases used, the proposed method, and the experimental design for evaluating it. The Results and Discussion sections present the most relevant information on the performance of the proposed method and its comparison with state-of-the-art methods. Lastly, the Conclusions section summarizes the most significant achievements, improvements, and future directions of the present research.

## 2. Related Works

Over the past decade, numerous approaches have been developed using digital image processing and machine learning techniques for automatic breast cancer detection and segmentation to aid experts in analyzing mammography images and providing a second opinion on possible abnormalities. For example, in  [26], a local geodesic ACM method was implemented for segmenting breast mass lesions on the Digital Database for Screening Mammography (DDSM). The results demonstrated the model’s effectiveness in identifying the radial and ambiguous edges of masses, yielding area-overlap (AOM) values of 0.85 and 0.87 for 4 mm and 6 mm radii, respectively. In  [27], an ACM based on local and global fitted functions was implemented to segment breast tumors on the mini Mammographic Image Analysis Society (MIAS) database, attaining scores of 0.984, 0.9802, and 0.985 for the accuracy (ACC), sensitivity, and DICE metrics. Moreover, in [28], Chan–Vese snakes and level sets were used for breast cancer detection in the mini MIAS database. The results highlighted the superior performance of the snake model, achieving an intersection over union (IoU) of 90%, a DICE score of 97%, and a precision (PRE) score of 95%.

Shallow learning models have also played a key role in classification tasks. For example, in [20], an SVM model was implemented with a structural risk minimization principle. A private dataset comprising 699 breast cancer samples was used to validate the model, yielding an accuracy (ACC) of 97.07%. In [21], an improved RF-based rule extraction method was developed to make classification rules for breast cancer diagnosis. It was evaluated on the Wisconsin Diagnostic Breast Cancer (WDBC), Wisconsin Original Breast Cancer (WOBC), and Surveillance, Epidemiology, and End Results (SEER) datasets, achieving an overall ACC of over 95%. In [22], an optimized kNN classifier was developed for breast cancer detection on the WBCD dataset, reaching an ACC score of 94.35%. Also, in [23], a breast cancer diagnostic method based on a DT model was proposed. It was tested on different training–test splits of the WBCD dataset, achieving an ACC of 94.3%. Moreover, in [24], a set of ANN models was implemented for breast cancer diagnosis, prognosis, and prediction. The models were tested on the WBCD dataset, and the best ACC of 95.82 was achieved by the learning vector quantization model.

Recently, deep learning models based on CNNs have emerged as robust feature extractors, serving as the backbone of several approaches for breast cancer analysis. For example, in [30], an optimized CNN model was proposed for detecting and diagnosing breast cancer using a raw dataset of 3895 thermal breast images, achieving an ACC score of 98.95%. A mass detection method based on deep CNNs and unsupervised extreme learning machine clustering was proposed in [31]. It was validated on a private dataset of 400 mammograms, achieving ACC and AUC values of 86.50% and 0.92, respectively. In [45], a deep CNN with an AlexNet model was implemented into a computer-aided detection (CAD) system and validated on the DDSM and the Curated Breast Imaging Subset of DDSM (CBIS-DDSM) databases, achieving an AUC of 0.94 and an ACC of 87.2%. In [46], four deep CNNs, GoogleNet, AlexNet, PSO-MLP, and ACO-MLP models, were implemented for detecting and classifying breast cancer lesions in the DDSM database. The best model was GoogleNet, which attained an ACC of 99%. Additionally, in [47], a seven-layer deep CNN was implemented to detect normal and abnormal mammogram lesions and was validated on the mini-MIAS database, achieving an ACC of 67%. Furthermore, an end-to-end Unet model that combines breast mass detection, segmentation, and classification in a single stage was proposed in [38] and evaluated on the CBIS-DDSM and INbreast datasets, achieving an IoU of 90.50% and an AUC of 0.99%. Similarly, in [39], attention and residual UNets, combined with atrous spatial pyramid pooling (ASPP), were developed for breast tumor detection and segmentation on a version of the DDSM database, on the INbreast, and a private database. Among the models, the residual Unet outperformed the others across the three databases with DICE scores of 89.52%, 95.28%, and 95.88%, respectively.

In contrast, some approaches have tackled the mass detection problem using overall object detectors. For example, in [35], a YOLO-based model was proposed to detect and segment masses on the DDSM and INbreast datasets. It achieved a true positive rate (TPR), mean average precision (mAP), F1-score, and IoU values of 95.7%, 65.0%, 74.5%, and 64%, respectively. Likewise, in [37], an end-to-end CAD system based on YOLO was proposed and validated on the InBreast dataset, reaching a detection ACC of 89.4%. In [41], a mask RCNN model was developed for lesion detection and classification. The model was validated on a private dataset of 307 ultrasound images, achieving an average PRE score of 0.75 and an overall ACC of 85%. Similarly, in [42], an automatic breast mass segmentation method based on a mask RCNN model was proposed. It was validated on the INbreast dataset, reaching PRE and F1 scores of 95.87 and 81.05, respectively. Finally, in [43], the fast CNN, faster RCNN, and RCNN models were compared for breast cancer detection and diagnosis on histopathological images from the Breast Cancer Histopathological Database (BreakHis), with faster RCNN achieving the highest performance, reaching an ACC of 84%.

Furthermore, transformer-based models have recently attracted attention for breast cancer diagnosis because they can capture long-range dependencies and contextual features. In [48], a hybrid model called EfficientUNetViT was proposed to segment breast cancer lesions by integrating a pretrained Vision Transformer (ViT) as encoder within a UNet architecture, combined with depthwise separable convolutions, and evaluated on mammograms from the BUSI ultrasound dataset, achieving PRE, REC, F1-score, IoU, and DICE values of 0.7901, 0.7882, 0.7584, 0.6292, and 0.7584, respectively. In [49], a pure transformer architecture was developed for classifying breast cancer lesions in mammograms from the DDSM, MIAS, INbreast, and VinDr datasets, achieving an average accuracy of 95.9% and AUC of 0.977. Additionally, a mass-detection method using Swin Transformers as the backbone, combined with CNNs via box fusion, was introduced in [50] and evaluated on a subset (OMI-H-MD) of the OMI-DB dataset, achieving a true positive rate (TPR) of 78.1% at 0.1 false positives per image (FPpIs).

Overall, the reviewed literature confirms that AI methods can support breast mass detection and segmentation in mammography, with particularly strong results reported for deep architectures using ROI-based or transfer-learning approaches. However, many published studies either rely on pre-centered lesion crops, focus solely on segmentation accuracy without explicit end-to-end lesion localization, or use architectures that require substantial computational resources [51]. These limitations present an opportunity for fully automatic pipelines that first localize candidate lesions and then refine their contours with lower computational demand, such as the proposed MassSeg-Framework.

## 3. Materials and Methods

### 3.1. Databases

**Figure 1:**
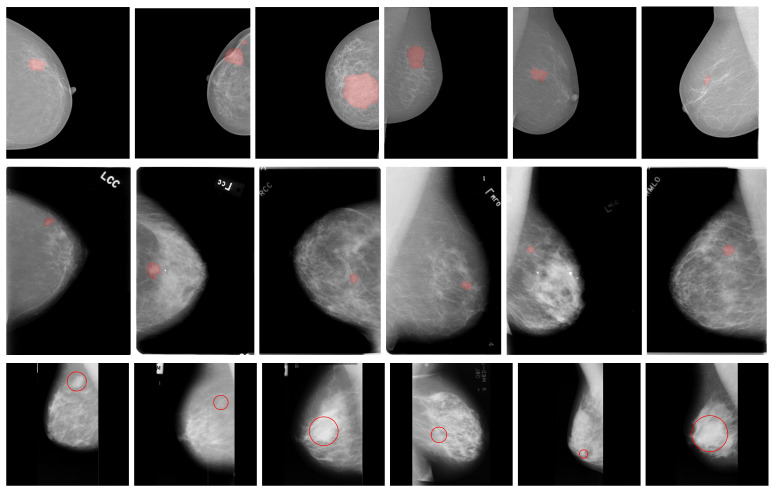
Randomly taken examples of mammography images and ground truth annotation (red area and circle) from the INbreast (first row), CBIS-DDSM (second row), and mini MIAS (third row) databases.

We considered three publicly available databases: the INbreast, the Curated Breast Imaging Subset of the Digital Database for Screening Mammography (CBIS-DDSM), and the mini MIAS. INbreast comprises 410 high-resolution full-field digital mammography images captured from craniocaudal (CC) and mediolateral oblique (MLO) projections. It includes annotations of findings, such as mass lesions based on ground-truth curve annotations and their corresponding expert assessments using BI-RADS and binary classifications [52]. Additionally, the dataset includes annotations for the pectoral muscle, speculated regions, distortions, clusters of microcalcifications, calcifications, and asymmetries [52]. CBIS-DDSM comprises 3103 scanned-film mammography studies with CC and MLO projections extracted from 1644 patient cases. It reports only calcification (753 samples) and mass (891 samples) lesions, based on expert-curated ground truth curve annotations [53]. The mini MIAS is composed of 322 scanned film mammography images containing MLO projections with 1024×1024 dimensions distributed in several classes such as calcification; well-defined/circumscribed masses; spiculated masses; other ill-defined masses; architectural distortion, asymmetry or normal; and severity, benign or malignant [54]. It also uses the ground truth based on the abnormality’s center point (x,y-coordinates) and establishes a radius to form a circle enclosing it. Examples of randomly selected mammography images with mass lesions and their corresponding ground truth annotations from the used datasets are shown in Figure 1.

### 3.2. Proposed MassSeg-Framework

**Figure 2:**
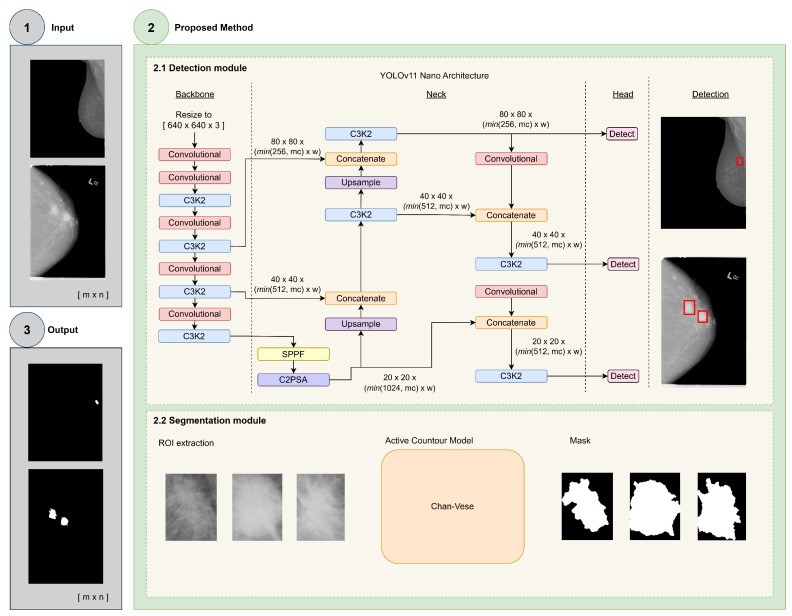
Workflow of the proposed *MassSeg-Framework*. First, the detection module identifies each lesion in the image and outputs the bounding boxes for each detection (red boxes). Subsequently, the detected boxes are fed into the segmentation module to produce the mask for each lesion.

The proposed approach is based on two modules, detection and segmentation, integrated into a single framework to build a two-step automatic breast mass detection and segmentation method, called *MassSeg-Framework*, for assessing mammography images, as shown in Figure 2.

The detection module implements the state-of-the-art YOLO (version 11) deep learning architecture to detect possible ROIs with a high probability of having mass lesions (see Figure 2, step 2.1). We selected the YOLOv11 architecture, the most advanced and stable release [55,56], over other object detectors, such as RCNN-derived models [41,42,43], due to its favorable cost–benefit trade-offs. Specifically, YOLOv11 operates as a single-shot, multi-scale detector, enabling object localization across different spatial resolutions in a single forward pass. It also incorporates the exponential linear unit (ELU) activation function, which mitigates vanishing gradients and accelerates convergence during training. Furthermore, the use of the generalized intersection over union (GIoU) metric, a more evolved version of the IoU function that takes into account the shape and dimensions of the bounding boxes, improves object localization precision and substantially reduces the model’s weight, making it more practical and efficient to deploy in limited-resource environments, where GPU memory, available compute, inference latency, or the need to process multiple studies simultaneously restrict the use of larger models. These features make it well-suited for improving time spent on the detection task while maintaining high accuracy.

YOLOv11 is composed of three main parts. The backbone performs the most feature extractions using several convolutional blocks. The neck integrates upsamples, concatenation, and convolutional operations for deeper feature extraction, and the head implements the classifier, providing the final detection and classification (Figure 2, step 2.1). Leveraging the overall structure of YOLOv11, most derived architectures modify the backbone (by increasing or decreasing the number of convolutional operations) and the head, adapting them to the problem to be solved.

The proposed method considered three YOLOv11 architectures, nano, small, and medium, which vary in complexity from simple to moderately complex. A detailed description of each of the considered architectures is provided below.

- *Nano*: The nano backbone begins with a single convolutional layer followed by a specialized block containing one convolutional and a C3K2 (Cross Stage Partial with smaller kernels of [3×3]) layer, which replaces the C2f (context to focus) layer used in previous YOLO versions. This block structure is repeated three times before connecting to the final SPPF (Spatial Pyramid Pooling) layer in the neck. This layer with a kernel size of [5×5] allows the model to effectively handle input images of different sizes and capture information at multiple scales [57]. Throughout the backbone, convolutional layers use a [3×3] kernel with a stride of 2- and 1-pixel padding, effectively extracting relevant image information while preserving spatial resolution and ensuring efficient downsampling. The C3K2 layer plays a crucial role in optimizing information flow through the backbone by improving feature representation using two lightweight convolutions instead of a single heavier one, reducing parameters and FLOPs while maintaining strong representational capacity compared with previous YOLO iterations’ C2f blocks [56]. In the neck component, two C3K2 layers feed into concatenation layers, enhancing multi-scale feature fusion [57].
- *Small*: The small backbone extends the nano architecture by adding 12 additional convolutional + C3K2 blocks, enabling the extraction of more features and contextual information from the input image, thereby improving the model’s capacity to handle complex visual patterns. Finally, as in the nano configuration, two C3K2 layers feed into other concatenated layers in the architecture’s neck.
- *Medium*: The medium backbone further expands the small architecture by increasing the number of feature-extractor blocks (convolutional + C3K2) to 48. This improvement adds 64 layers to this part of the architecture, enabling the model to explore and exploit more information from the input image and to perform deeper hierarchical feature extraction, resulting in more precise detection in dense or detailed visual environments [57]. Similar to the nano and small backbones, two C3K2 layers connect to other concatenated layers in the neck part of the architecture.

For all architectures, the neck and head parts remain constant. The neck consists of four C3K2 and concatenation layers, followed by two upsampling layers and convolutional layers that match the backbone configuration. Additionally, YOLOv11 distinguishes itself by incorporating the C2PSA (Cross Stage Partial with Spatial Attention) mechanism, which enhances the model’s ability to focus on spatially significant features like small or occluded objects [56,57]. This mechanism extracts additional features that enhance information representation and facilitate multi-scale feature fusion [55,56]. The head (classifier) part contains three detection blocks. Each implements C3K2 layers that refine features at different depths and spatial resolutions, following the same structural configuration as in the backbone and neck. These blocks also incorporate CBS (Convolution–BatchNorm–SiLU) layers to improve normalization and gradient flow. Finally, each block ends with a series of 1×1 and 3×3 convolutional layers—including final 1×1 convolutions with stride 1 and zero padding—to further process the aggregated features before passing them to the bounding-box and class prediction layers responsible for computing object localization and class probabilities [55,56].

The segmentation module uses a Chan–Vese ACM to segment the mass lesion within the ROIs provided by the detection module (see Figure 2, step 2.2). An ACM is a deformable curve defined in a continuous domain whose evolution is governed by external forces derived from the image data and internal forces imposed by the model [58]. The external forces guide the active contour towards the object’s contours presented in the images, and the internal forces act as a smoothness constraint [59]. We chose the Cha-n-Vese model over alternatives, such as the Geodesic model [60], because it minimizes the Mumford–Shah functional, enhancing its ability to handle variations in object appearance. It avoids relying directly on image gradients and is therefore suitable for low-contrast lesion boundaries. Thus, it is more robust for segmentation tasks in complex scenarios, such as breast mass segmentation [61], reducing the problem of local minima in the data. Its energy functional is defined as follows:

(1) $$\begin{array}{cc} F(c_{1},c_{2},\phi ) & =\mu \int_{\Omega }\delta \left(\phi \left(x,y\right)\right)\left|\nabla \phi (x,y)\right|dxdy\\ & \;+\nu \int_{\Omega }H\left(\phi \left(x,y\right)\right)dxdy\\ & \;+\lambda_{1}\int_{\Omega }{\left|u_{0}\left(x,y\right)-c_{1}\right|}^{2}H\left(\phi \left(x,y\right)\right)dxdy\\ & \;+\lambda_{2}\int_{\Omega }{\left|u_{0}\left(x,y\right)-c_{2}\right|}^{2}(1-H\left(\phi \left(x,y\right)\right)dxdy \end{array}$$

where $C_{1}$ and $C_{2}$ are constants given by the average of $u_{0}$ inside and outside the region delimited by the zero level set of ϕ. *H* is the Heaviside (unit) step function and μ, ν, $\lambda_{1}$ and $\lambda_{2}$ are contants, usually $\lambda_{1}$ = $\lambda_{2}=1$ and ν=0.

### 3.3. Experimental Setup

This section outlines the experimental methodology employed to train and validate the proposal. Thus, some of the necessary procedures to reach the goal are described in detail below.

#### 3.3.1. Experimental Dataset Creation

The databases used contain several findings. We considered only the mass lesions, which are the target of this work, to create the experimental datasets (one per database). Additionally, eighteen images from the CBIS-DDSM database were removed due to inconsistencies in the expert-annotated ground truth lesions, as reported in [62]. Therefore, the *dINBreast* dataset consists of 115 mass lesions extracted from 106 mammography images of the INbreast database, the *dCBIS* dataset contains 1686 mass lesions from 1582 mammography images of the CBIS-DDSM database, and the *dMIAS* dataset comprises 58 mass lesions from 55 mammography images in the mini MIAS database.

#### 3.3.2. Image Preprocessing

All images were resized to 640×640 pixels to reduce the input space while preserving the mass lesion contour details and meeting the input size requirements of the employed YOLOv11 architectures [55]. Then, after the detection step, all detected bounding boxes (cropped ROIs), which corresponded to the predicted YOLOv11 bounding box in the resized 640×640 mammogram image, were preprocessed using the contrast-limited adaptive histogram equalization (CLAHE) operation and a median filter with a [3×3] kernel size to enhance the image quality, reduce noise, and preserve the object’s boundaries. Here, each ROI corresponded to the predicted YOLOv11 bounding box in the resized 640×640 mammogram image.

#### 3.3.3. Training, Validation, and Test Set Creation

We used training–test split ratios of 90%–10% and 70%–30% for the *dINBreast* and *dCBIS* datasets, respectively. These ratios are traditional splits and were established based on the image counts for each experimental dataset, ensuring successful training of the implemented models. The training partition feeds the stratified 10-fold cross-validation method to train and validate the YOLOv11 architectures in the detection module, and the test partition remains outside the training process to assess their generalization capability in the presence of similar data [63]. Moreover, we use the *dMias* dataset as an additional external test set to evaluate its robustness across different datasets.

#### 3.3.4. Training Parameter Optimization

To optimize the training performance of the YOLOv11 architectures, we conducted a preliminary hyperparameter search procedure using the Ray Tune library with the Asynchronous Successive Halving Algorithm (ASHA). This strategy allowed us to efficiently explore a predefined search space while allocating computational resources to the most promising configurations [64]. In this way, minimizing computational costs during training.

The search was conducted individually for each experimental dataset. In this tuning process, we selected key hyperparameters based on their known influence on training stability and performance: initial learning rate (lr0), final learning rate factor (lrf), weight decay (weight_decay), box loss weight (box), and classification loss weight (cls), as well as data augmentation parameters including thescale factor (scale), translation factor (translate), and mosaic probability (mosaic). The ranges were defined empirically as follows: $lr0\in [5\times 10^{-5},5\times 10^{-3}]$, lrf∈[0.05,0.5], $weight\_decay\in [5\times 10^{-5},5\times 10^{-4}]$, box∈[0.02,0.12], cls∈[0.3,1.5], scale∈[0.1,0.4], translate∈[0.0,0.15], and mosaic∈[0.1,0.5].

The YOLOv11n model served as the base configuration for the tuning phase, with 50 training epochs for each trial. All experiments were executed on a single L4 GPU, with GPU settings configured to maximize performance within the Colab environment. The best-performing configuration to train the final models for each cross-validation fold is shown below.

*dCBIS*: {

lr0: 0.000480736,

lrf: 0.157264,

weight_decay: 0.000318486,

box: 0.0473327,

cls: 0.794108,

scale: 0.311104,

translate: 0.0104767,

mosaic: 0.456487

}

*dINbreast*: {

lr0: 0.000675362,

lrf: 0.0582257,

weight_decay: 0.000142265,

box: 0.0917594,

cls: 1.31841,

scale: 0.257247,

translate: 0.0175932,

mosaic: 0.304154

}

Additionally, during training, multi-scale augmentation was enabled, which randomly resizes input images within a predefined range. This strategy improves the model’s ability to generalize across varying resolutions and anatomical variations, which is particularly beneficial in medical imaging, where lesion sizes and shapes can vary significantly. Given the clinical importance of accurately detecting masses without redundant or missed predictions, the Intersection over Union (IoU) threshold was set to 0.5 during validation. This value directly affects Non-Maximum Suppression (NMS), a crucial post-processing step that reduces multiple overlapping detections of the same lesion, thus improving the reliability of the results. Finally, the maximum number of detections per image was set to 10 to enable comprehensive scanning of dense breast tissue without overwhelming the model.

#### 3.3.5. *MassSeg-Framework* Configuration

For training the YOLOv11 architectures (nano, small, and medium) in the detection module, the number of epochs was set to 200, with a callback function to monitor and select the best model, and the batch size was set to 16. The model was trained using the Adam optimizer with the training parameters described in the previous section. The confidence threshold ranged from 0.4 to 0.9 with steps of 0.1, providing a reasonable confidence analysis.

To implement the Chan–Vese active contour model in the segmentation module, we used μ=3, $\lambda_{1}=\lambda_{2}=1$ and ν=0, together with an initial region defined as a circle centered at the (X,Y) coordinates of predicted YOLOv11 bounding box (ROIs) in the resized 640×640 mammogram image, with a radius of 40 pixels and 436 iterations. The μ parameter was empirically determined to balance contour regularization and adherence to lesion boundaries. The radius and iterations were chosen following previous research [61], where these parameters were optimized for the same problem.

#### 3.3.6. Validation Metrics and Selection Criteria

We computed different validation metrics to assess the proposed *MassSeg-Framework*. For the detection step, the mean and standard deviation of PRE, REC, and mAP at an IoU of 0.5 (mAP50) were calculated. The latter was considered the primary metric for discussing the results, as it is based on precision and recall across different confidence thresholds, providing a more comprehensive view of model performance [65]. Additionally, for the segmentation step, we used the mean DICE [66] metric instead of the mean IoU [67] to evaluate segmentation quality. Both metrics evaluate the segmentation’s accuracy relative to the ground truth. However, the DICE metric is used to evaluate the results because it is more sensitive to smaller objects, such as breast cancer lesions, and it avoids class bias by treating false positives and negatives equally [68].

Furthermore, we calculate the 95th percentile Hausdorff Distance (HD95) as a boundary-based metric to supplement the DICE results. The Hausdorff Distance measures the distance between the predicted and reference contours, and using the 95th percentile reduces sensitivity to outliers and noise. Due to variability in image resolution across datasets and the fully automatic nature of the proposed framework, the median and mean of HD95 scores offer a reasonable evaluation of segmentation performance. It should be noted that this metric was calculated in pixel units (px), since only the *dINbreast* dataset provides DICOM-based spatial resolution, while *dCBIS* consists of scanned film mammograms without consistent physical calibration.

Although there is no golden rule for selecting the best model in the detection module, we first considered the model that maximizes mAP50. Second, if models achieve mAP50-tied performance statistically comparable to other architectures, we preferred the one with the lowest computational cost (i.e., nano, small, and medium, in that order).

## 4. Results and Discussion

As described in the experimental setup section, the proposed *MassSeg-Framework* was trained and validated at confidence thresholds ranging from 0.4 to 0.9 on both experimental datasets using stratified 10-fold cross-validation. Then, we tested it on external test sets and on the entire *dMIAS* dataset as a holdout set, which was not used during training. A summary of the results for validation metrics and statistical comparisons is presented in Table 1 and Table 2.

### 4.1. Performance of the Detection Module

In the *dINBreast* breast mass detection experiment (see Table 1), across all models, the optimal operating point occurred at the lowest tested threshold of 0.4, where the mean mAP50 is maximized, achieving 0.862, 0.849, and 0.843 for the YOLOv11 nano, small, and medium, respectively. This suggests that a significant portion of true mass detections are detected with moderate confidence scores. Hence, applying stricter thresholds gradually filters out true positives and diminishes overall detection quality.

The YOLOv11-nano model provided the most stable and strongest performance across thresholds, with a high mean mAP50 of 0.862±0.06, a high mean PRE of 0.952±0.06, and the highest mean REC of 0.769±0.11 at a threshold 0.4. This balance is especially suitable for mammography screening, where missing lesions can be costly. Even as the threshold rises, this model maintains a relatively high mean PRE around 0.95–0.97 and degrades more smoothly than larger models, with a mean mAP50 dropping from 0.862 at 0.4 to 0.587 at 0.9. At the most conservative setting with a threshold of 0.9, it achieved a perfect mean PRE of 1.000, but the mean REC drops to 0.174, indicating a very strict detection approach that minimizes false positives but increases missed masses.

The YOLOv11-small model demonstrated high mean PRE at low-to-mid thresholds, e.g., 0.980 at 0.4 and 0.978 at 0.6, but showed a sharper decline in mean REC and mAP50 as the threshold increased. Beyond 0.6, performance dropped quickly, as demonstrated by the mean mAP50 of 0.820 at 0.5 to 0.181 at 0.9, with the mean REC falling to 0.061 at 0.9. This indicates that the small architecture is more affected by confidence filtering, resulting in sparse, less reliable predictions at high thresholds and making it less suitable for high-sensitivity clinical settings when a conservative threshold is used.

The YOLOv11-medium model generally maintained a better mean REC than YOLOv11-small at mid-range thresholds, e.g., 0.646 at 0.6 and 0.472 at 0.7, but it still did not outperform YOLOv11-nano’s mean mAP50 at any threshold. The YOLOv11-medium’s performance also dropped significantly at high thresholds, reaching a mean mAP50 of 0.140 and REC of 0.080 at 0.9. Overall, this architecture had some recall benefits over the small architecture at intermediate thresholds, but it remained less robust and less accurate than the nano in overall detection quality.

The reported statistical comparison of mean mAP50 in this dataset showed that as thresholds move farther from the optimal range of 0.4–0.5, performance differences become increasingly statistically significant (*p* < 0.05). This confirmed that threshold choice significantly affects mammography mass detection and that YOLOv11-nano operating at 0.4 was the best choice in this dataset in accordance with the stated selection criteria.

Across all models in the *dCBIS* dataset (see Table 2), the best confidence threshold is at the lowest tested value (0.4), at which the mean mAP50 is maximized for the nano (0.709), small (0.681), and medium (0.704) architectures. This pattern suggests that a considerable fraction of true mass detections are assigned moderate confidence scores. Consequently, increasing the threshold progressively removes true positives, reducing recall and degrading overall detection performance.

The YOLO-nano model (winner) provided the most solid performance across thresholds, achieving the best balance at 0.4 with a mean mAP50 = 0.709 ± 0.03, a mean PRE = 0.778 ± 0.05, and a mean REC = 0.601 ± 0.07. This operating point is particularly relevant for mammography screening, where sensitivity is critical and missed masses are tragic. As the threshold increases, nano’s mean PRE score improves steadily, reaching 0.953 at 0.8, but the mean REC declines sharply from 0.601 (0.4) to 0.033 (0.9), and the mean mAP50 decreases from 0.709 (0.4) to 0.404 (0.9). At the most conservative threshold of 0.9, the nano architecture maintains moderate precision (0.775) but exhibits very low recall (0.033), reflecting a selective process that limits false positives at the expense of missing most lesions.

The YOLO-small architecture demonstrates competitive precision at moderate thresholds (e.g., 0.849 at 0.6, 0.901 at 0.7) but suffers a sharper deterioration in both mean REC and mAP50 as the threshold increases. Its mean mAP50 score drops from 0.681 (0.4) to 0.176 (0.9), while the mean REC falls from 0.574 to 0.010, indicating that high thresholds yield too few surviving detections for reliable mass identification. Moreover, at strict threshold filtering (0.8–0.9), this architecture becomes less dependable (e.g., mean PRE = 0.342 ± 0.43 at 0.9), suggesting unstable behavior when only very high-confidence predictions are retained.

The YOLO-medium architecture’s performance is close to that of the nano at low thresholds (e.g., mean mAP50 = 0.704 and mean REC = 0.591 at 0.4), but it does not surpass the nano’s best mean mAP50 and becomes increasingly conservative as the threshold rises. The medium architecture’s recall declines from 0.591 (0.4) to 0.003 (0.9) and its mAP50 drops from 0.704 to 0.051, showing the strongest collapse at the strictest threshold among the three. While this architecture can offer slightly higher PRE at some mid-to-high thresholds (e.g., 0.949 at 0.7), it entails a substantial loss of sensitivity, which is unfavorable for screening-oriented detection.

The U-test results in this dataset indicate that performance differences become statistically significant as thresholds move away from the optimal region. Across all architectures, p-values are frequently < 0.05 from 0.6 onward, indicating that threshold selection materially affects detection performance. Overall, these findings support the use of low thresholds (0.4–0.5) for mammography mass detection, as they better preserve clinically important true positives, whereas thresholds at or above 0.8 should generally be avoided because they substantially reduce recall and yield overly conservative detectors that miss most lesions.

The difference in detection performance between the two experimental datasets is related more to differences in data quality rather than model’s learning. The *dINbreast* dataset contains high-quality, full-field digital mammography images, providing more precise details of breast characteristics, making it easier to distinguish between breast tissue and potential mass lesions and reducing the effort required for detection models to achieve good results. That explains why several models from different YOLOv11 architectures achieved good detection performances. In contrast, the *dCBIS* dataset comprises scanned film mammography studies, which significantly degrade image quality. High-density breasts, together with suboptimal image quality, obscure the details of breast lesions, such as the mass’s texture and contour, which become confused with the surrounding breast tissue, making it harder for the models to process less evident mass characteristics and complicating their learning.

### 4.2. Training Dynamics and Computational Footprint of YOLOv11 Nano

**Figure 3:**
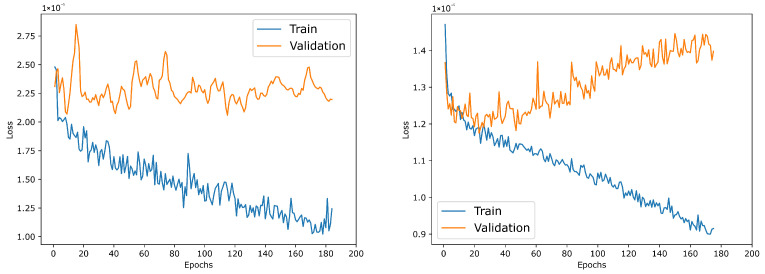
Learning process of the best-selected YOLOv11 nano architecture for the *dINbreast* (**left**) and *dCBIS* (**right**) datasets.

Both winning models converge rapidly and remain stable on both mammography datasets, with the training loss decreasing smoothly across epochs, as shown in Figure 3. For the *dINbreast* dataset, the training loss steadily declines, whereas the validation loss remains consistently higher and exhibits a barely perceptible zigzag pattern (small oscillations) after the initial epochs (see Figure 3, left plot). This persistent generalization gap suggests that, despite effective optimization, validation performance remains sensitive to sample composition and lesion variability, consistent with limited training data and high inter-case heterogeneity in mammography. Nonetheless, the validation curve does not diverge, indicating no overfitting but a regime in which additional epochs mostly improve the training fit without proportionally reducing the validation loss.

On the other hand, on the *dCBIS* dataset, both training and validation losses reach lower values and remain closer together throughout training, indicating a more stable and consistent learning process (see Figure 3, right plot). After the early convergence phase, the validation loss shows a slight upward drift while the training loss continues to decrease, a pattern characteristic of apparent overfitting in later epochs. However, the effect is small, and the curves remain well-behaved.

Overall, these plots support the conclusion that YOLOv11-Nano can be trained reliably for breast mass detection while highlighting that dataset properties strongly influence generalization. For example, the *dINbreast* dataset shows greater sensitivity in validation loss, likely due to its smaller dataset size and the subtle appearance of lesions, whereas the *dCBIS* dataset exhibits smoother optimization dynamics. From a clinical deployment perspective, these trends motivate using cross-validation, early stopping, regularization, and augmentation to maximize generalization, particularly on smaller, high-quality datasets, where subtle findings can lead to higher variance in validation performance.

Regarding hardware profiling, in the *dINBreast* dataset (see Table 3), there is a clear efficiency gap among the YOLOv11 variants. The nano architecture was the lightest model (3.307 GFLOPs, 2.624 M parameters) and requires the lowest compute during learning and evaluation, with 275.28±19.37 s of training time, around 5.0 GB of GPU memory, and 280.34 s of test time at approximately 4.94 GB. Demand increases noticeably for the small (10.859 GFLOPs, 9.459 M parameters) and medium (34.264 GFLOPs, 20.115 M parameters) architectures. The small architecture increases the memory demand close to 9.28 GB and the test time to 406.47 s, while the medium architecture roughly doubles the small architecture’s compute again, reaching 682.93±95.15 s training time and nearly 17.7 GB memory with 794.11 s of test time. These results indicate that the time plus memory footprint scales steeply with model size. For mammography mass detection, where systems must be responsive and deployable on constrained clinical hardware, lower-compute detectors are generally preferable as long as accuracy is maintained because they reduce inference latency, GPU requirements, and operational cost.

The same scaling trend holds for the more demanding *dCBIS* dataset (see Table 4). The nano architecture maintains the smallest model complexity (3.307 GFLOPs, 2.624 M parameters) and the lowest memory use (approximately 5.0 GB), while the small and medium architectures require substantially more memory, around 9.2 GB and 17.7 GB, respectively, and extended the training time to 2793.96±444.17 s for the small architecture and 5405.33±565.79 s for the medium architecture, compared to the nano (2177.08±225.22 s). The time consumed during the test is also high across models, with the medium architecture remaining the most expensive (6411.78 s), whereas the nano and small architectures are comparable at 3591.89 s and 3043.72 s, respectively.

In general, across both datasets, the nano architecture offers the most favorable efficiency profile with the lowest FLOPs/parameters, memory footprint, consistently fast training, and competitive test–time cost, which is typically preferable in mammography workflows where, in addition to detection performance, scalability and deployment feasibility are critical. These differences are clinically relevant not because larger models are impossible to run on GPU workstations but because lower memory and computation requirements make the framework easier to deploy across multiple acquisition points, scale in centralized screening workflows, and operate with lower latency and infrastructure costs.

### 4.3. The MassSeg-Framework Performance on the Test Sets

**Figure 4:**
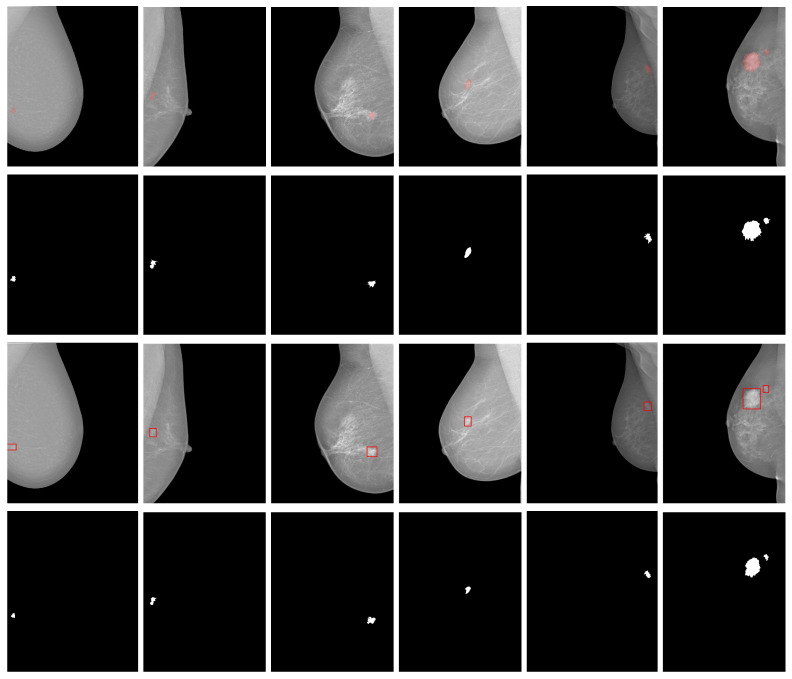
Examples of the *MassSeg-Framework* architecture’s performance on six randomly selected cases (one benign and five malignant) from the test set of the *dINbreast* dataset: original image with the ground truth annotation (first row, red area), ground truth mask (second row), detected ROIs with mass lesions (third row, red boxes), and the final segmentation (fourth row).

**Figure 5:**
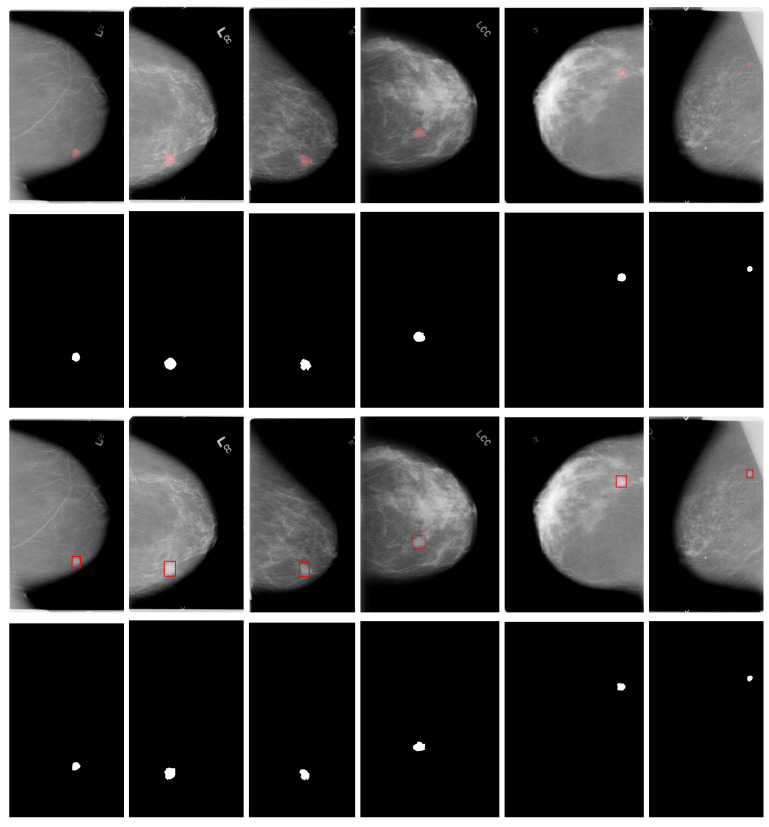
Examples of *MassSeg-Framework*’s performance on six randomly selected cases (two benign and four malignant) from the test set of the *dCBIS* dataset: original image with the ground truth annotation (first row, red area), ground truth mask (second row), detected ROIs with mass lesions (third row, red boxes), and the final segmentation (fourth row).

We first integrated the best-ranking models from the optimization phase into the detection module of the proposed *MassSeg-Framework*. Then, we evaluated the entire method on holdout sets to assess the quality of the proposed end-to-end framework, as shown in Figure 4 and Figure 5. From these figures, it is evident that the detector consistently localizes mass regions with compact bounding boxes, enabling the subsequent segmentation stage to operate on a reduced search space and limiting interference from irrelevant background structures.

In terms of detection performance on the *dINbreast* test set, the proposed *MassSeg-Framework* achieved an outstanding mean mAP50 of 0.909, demonstrating strong performance on this task. Moreover, these results can be corroborated visually on random examples (one benign and five malignant), which highlight the method’s ability to identify lesions across varying mass sizes (see Figure 4, columns 1, 2, and 3) and locations (see Figure 4, column 1). Most examples are small, subtle findings with limited contrast that were nevertheless detected, indicating that the model captures discriminative cues beyond strong intensity differences. In addition, lesions located near the breast boundary or embedded in heterogeneous parenchyma (challenging anatomical regions) were successfully localized, suggesting robustness to positional variability. Cases with a cluttered background and a bit of breast density were detected with particular accuracy (see Figure 4, columns 1 and 3), while maintaining plausible localization, implying that the learned representation can separate true lesions from confusing dense patterns. There was a special case involving two breast mass lesions, where the proposed method successfully detected both (see Figure 4, column 6), even though it was trained on a small number of multi-breast mass lesions. This result highlights the model’s detection performance on this dataset.

Regarding the segmentation module, it successfully extracted each lesion while preserving its contour, as evidenced by a mean DICE score of 0.721, and HD95 median and mean values of 45.83 px and 535.77 px, respectively. These results are substantial and indicate strong spatial agreement with the ground truth annotations. The low median indicates that, in most cases, the predicted contours closely match the ground truth boundaries, confirming the robustness of the segmentation module. However, the higher mean score is influenced by a small number of challenging cases expected in complex mammographic scenarios. Preserving the lesion core and contour is vital for subsequent severity assessment and diagnosis. For example, ill-defined mass shapes with irregular contours (e.g., micro-lobulated, lobulated, spiculated) are symptoms of malignancy, as shown in Figure 4, columns 1, 2, 4, 5, and 6. In contrast, well-defined shapes and rounded contours are always benign symptoms, as shown in Figure 4, column 3. These results were expected, as the best matches occur when the lesion exhibits a more cohesive intensity transition, whereas minor discrepancies become apparent in cases with irregular margins and weak lesion contrast, where boundary ambiguity is expected even for expert annotation.

For the test set of the *dCBIS* dataset, the proposed framework successfully detected all breast masses for a mean mAP50 score of 0.90, demonstrating consistent end-to-end behavior, since the detector successfully produces compact, well-placed bounding boxes around the suspicious regions, and the further ACM stage refines these ROIs into coherent lesion masks, confirming that the proposed pipeline can localize and segment masses under diverse imaging conditions.

From Figure 5, it is possible to observe that the randomly selected six examples (two benign and four malignant) illustrate robustness to clinically relevant variability in mass size, location, and surrounding tissue texture. Tiny masses, which are more difficult to detect on lower-quality mammography images, were found without hesitation (see Figure 5, columns 1, 5, and 6). Additionally, mass lesions within dense breast tissue (complex fibroglandular texture) were correctly detected (see Figure 5, columns 3 and 4). Despite cluttered backgrounds that can increase false alarms, the detector retains focused ROIs, highlighting the importance of the proposed approach. This suggested that YOLOv11 nano captures discriminative features of breast masses beyond simple intensity contrast, including features related to anatomical location, lesions near the breast boundary, and lesions within denser central tissue regions, where background structures and compression artifacts can mimic mass-like patterns.

Regarding segmentation, the ACM-based refinement achieved a mean DICE score of 0.700, indicating strong agreement between the predicted masks and the ground truth annotations. The HD95 median value of 132.85 px and the mean of 991.33 px indicate consistent contour alignment in most cases. The gap between these metrics highlights the influence of a small subset of more challenging instances, as in the previous dataset. The strongest matches occurred for well-defined masses, whereas small deviations in boundary shape due to lesion margins partially obscured by dense tissue were expected to affect the performance, a common challenge in the *dCBIS* dataset.

Overall, the proposed *MassSeg-Framework* is effective for mammography mass analysis, providing reliable candidate localization across diverse imaging conditions on both datasets. The combination of DICE and HD95 metrics highlights that the method achieves strong agreement with expert annotations in most cases while also revealing limitations in challenging scenarios. Furthermore, the ACM-driven segmentation produces lesion contours that closely follow expert masks, including in dense breast scenarios where both detection and boundary definition are inherently challenging. While these results demonstrate robust retrospective algorithmic performance, they should not be interpreted as evidence of clinical efficacy without reader studies or prospective validation.

### 4.4. The MassSeg-Framework Qualitative External Evaluation

**Figure 6:**
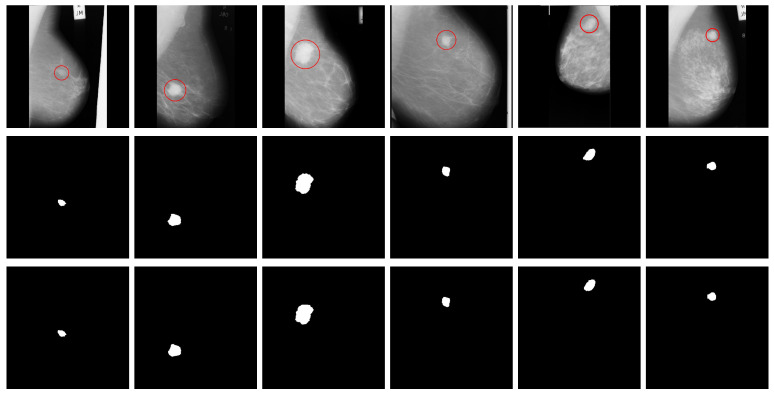
Examples of *MassSeg-Framework*’s performance on six randomly selected cases (three benign and three malignant) from the *dMIAS* dataset: original image with the ground truth annotation (first row, red circle), the resultant masks when trained with the *dINbreast* (second row) and *dCBIS* (third row) datasets, respectively.

We also validated the proposed framework’s generalization in a cross-dataset setting for mass detection and segmentation on the *dMIAS* dataset. Note that the ground truth annotation in this dataset consists of a point and a radius, which together define a circle that contains the mass lesion. This type of annotation limits the creation of reliable ground truth masks for breast masses, hindering quantitative comparisons with the segmented masses and the computation of the DICE metric. In this sense, we considered segmentation successful when the mass area was fully extracted, and the mass contour corresponded to the lesion’s severity (benign or malignant), as annotated by experts. Hence, we applied the *MassSeg-Framework*, trained on the *dINbreast* and *dCBIS* datasets, to the external *dMIAS* dataset, and the resulting detection and segmentation performances are shown in Figure 6.

From a detection viewpoint, the qualitative examples suggest successful localization despite variability in lesion size and position. Some cases depict small, compact findings as well as more conspicuous masses, and the predicted regions remain spatially consistent with the annotated ground truth (circles), even when lesions occur near the breast boundary or within dense tissue. This is important because *dMIAS* images differ from *dINBreast* and *dCBIS* images in resolution and contrast, and such domain shifts often degrade lesion localization. The fact that predicted regions still fall within the ground truth annotations supports the framework’s ability to learn transferable cues for mass appearance rather than overfitting to dataset-specific intensity patterns.

As shown above, the proposed framework satisfactorily segments all breast mass lesions while preserving their shapes and contours independently of the training basis. All benign masses retained a well-rounded shape and regular contours, indicating successful segmentation performance (see Figure 6, columns 1, 2, and 5). On the other hand, excepting one malignant case where the *MassSeg* method trained on the *dINbreast* highlighted a down peak of segmentation versus an up peak encountered by the *MassSeg* method trained on the *dCBIS* (see Figure 6, column 6), the remaining cases were correctly extracted (see Figure 6, columns 3 and 4). Despite the trained model’s segmentation decision, the extracted mass exhibited an irregular contour, a feature of malignancy confirmed by the ground truth data. Therefore, regardless of whether the training weights were obtained from *dINbreast* or *dCBIS*, the proposed framework successfully segments all cases under domain shifts, which is a significant achievement.

### 4.5. State of the Art Comparison

Several deep learning-based approaches have achieved outstanding segmentation performance for mass lesions in mammograms, as shown in Table 5.

In the *dINBreast* dataset, sucessfull scores have been reported by Dhungel et al. (0.88) using deep structured learning with cropped ROIs [69], by Oliveira et al. (0.83) using ResNet50+VGG16+shortest-path on graphs with transfer learning and augmentation [70], and for ROI-based deep architectures such as the multi-level nested pyramid network of Wang et al. (0.91) [71], Baccouche et al. with Connected-UNets (0.95) [39], Alkhaleefah et al. with Connected-SegNets (0.96) [72], and the U-Net variants of Soulami et al. (0.99 with 128 × 128 and 256 × 256 inputs) [38]. On the other hand, in the *dCBIS* dataset, high DICE values are reported by Wang et al. (0.91) [71], Baccouche et al. (0.89) [72], Alkhaleefah et al. (0.93) [72], and Ahmad et al. with Associated-ResUNets (0.95) [73]. In comparison, the proposed *MassSeg-Framework* achieves values of 0.72 (*dINBreast*) and 0.70 (*dCBIS*) while using automatic detection on the resized image and a substantially larger input size of 640 × 640.

It should be noted that this comparison highlights a complementary strength of the proposed approach. While many top-scoring methods are trained and evaluated on cropped ROI-based inputs as in [69,71,72], and exploit transfer learning and data augmentation as in [70], the *MassSeg-Framework* targets a more realistic end-to-end setting by integrating detection and segmentation without relying on such training dependencies. In practical mammography workflows, segmentation quality is constrained not only by boundary determination but also by whether the lesion is correctly localized in the first place. Therefore, ROI-based pipelines can report higher DICE partly because they start from lesion-centered crops that reduce background complexity and ambiguity. Under this more challenging, fully automatic scenario, the proposed method demonstrates that clinically meaningful mass segmentation is achievable directly from detected regions at higher resolution, and the reported DICE score provides a conservative estimate of segmentation fidelity when localization uncertainty is accounted for. This result suggests that future improvement for the *MassSeg-Framework* could be achieved by incorporating data augmentation and transfer learning, strategies shown to be beneficial in Oliveira et al. [70] and other deep pipelines, while preserving its key comparative advantage, a unified and fully automatic detection and segmentation framework suitable for deployment on full-field mammography. Another promising direction is to extend the current Chan–Vese stage by incorporating more advanced active contour formulations revisited in [74,75], which better incorporate boundary, texture, and shape priors. Therefore, the main improvement offered by *MassSeg-Framework* is more methodological and practical than purely numerical. It enables end-to-end lesion localization and segmentation within a more realistic workflow, with lower computational requirements than many other deep segmentation pipelines.

### 4.6. Clinical Impact

The proposed *MassSeg-Framework* shows potential as a technically efficient tool for automated breast mass analysis and clinical decision support in breast cancer management. First, it achieves successful mass detection and segmentation with reduced computational complexity, making it a promising solution for settings where GPU memory, inference time, or throughput for large-scale screening make lighter architectures advantageous. This capability is particularly relevant in regions where access to advanced computational infrastructure is limited, yet reliable diagnostic tools are critical. Second, its scalability is a valuable tool for large-scale mammography screening programs. It could facilitate rapid screening in high-throughput environments, such as national breast cancer screening initiatives, by reducing the computational burden while maintaining accuracy. This scalability and its demonstrated ability to handle low-quality mammograms (e.g., results on the *dCBIS* dataset) highlight its robustness in real-world clinical scenarios. Third, it demonstrates an ability to preserve lesion contours during segmentation, a clinically significant feature. Lesion contours are critical for differentiating benign from malignant masses and for guiding follow-up diagnostic procedures. For example, the segmentation results in the *dINBreast* dataset preserved the irregular contours of malignant masses while maintaining well-defined boundaries for benign cases, aligning with the BI-RADS classification system used in clinical practice. Such performance may assist radiologists by preserving lesion contour information relevant to interpretation. However, its effect on diagnostic accuracy, recall decisions, and biopsy recommendations was not evaluated in this study. However, the present study evaluates computational efficiency rather than healthcare-system cost-effectiveness. Therefore, no formal economic analysis of implementation, workflow, medical personnel, or downstream diagnostic costs is claimed.

Finally, integrating the *MassSeg-Framework* into clinical workflows could enhance radiologists’ efficiency by providing an automated second opinion. Previous studies have shown that artificial intelligence systems can detect up to 20% more cancers than manual double reading [19]. Thus, its deployment in underserved populations could help bridge the gap in access to quality breast cancer diagnostics, although its effect on patient outcomes and radiologist performance remains to be established in reader studies and prospective clinical evaluations.

## 5. Conclusions

This work introduces the *MassSeg-Framework*, a fully automatic two-stage pipeline for breast mass analysis in mammography that integrates YOLOv11-based detection with Chan–Vese ACM refinement to achieve accurate mass localization and segmentation with a lightweight computational footprint. The framework was trained and evaluated on two publicly available datasets (*dINBreast* and *dCBIS*) using consistent experimental protocols. In the detection stage, YOLOv11-nano emerged as the most effective architecture, with an optimal confidence threshold of 0.4, achieving statistically significant mAP50 values of 0.862 and 0.709 on the *dINbreast* and *dCBIS* datasets, respectively. These results confirm that a moderate threshold preserves clinically relevant true-positive candidates, which is particularly important for screening-oriented settings where missed lesions are costly. In the segmentation stage, the proposed framework achieved mean DICE, median HD95, and mean of HD95 scores of 0.721, 45.83 px, and 535.77 px, and 0.700, 132.85 px, and 991.33 px on the test sets of the same datasets, demonstrating consistent overlap with expert annotations after automatic ROI detection and supporting the feasibility of end-to-end mass segmentation without manual cropping. These results support the technical feasibility of the proposed framework, but they do not establish clinical efficacy, radiologist performance improvement, or patient outcome benefits.

Compared with state-of-the-art approaches that commonly assume lesion-centered ROIs or rely on heavier backbones, the proposed pipeline addresses a more realistic scenario by performing automatic detection followed by segmentation while maintaining substantially lower computational requirements. This balance between performance and efficiency makes the *MassSeg-Framework* a promising component for scalable mammography analysis, particularly in high-throughput screening workflows or in deployment settings where a lower memory footprint, lower latency, and reduced hardware requirements are advantageous. Its principal contribution is thus not only segmentation accuracy but also the integration of automatic detection and local contour refinement within a lightweight end-to-end framework. Although the framework demonstrated favorable computational efficiency, this should not be interpreted as a formal health-economic evaluation.

Future work will focus on refining the image preprocessing steps to further enhance segmentation quality, particularly for low-quality mammograms, by evaluating more advanced detector architectures, such as YOLOv12, to assess their performance in both experimental datasets and exploring more advanced active contour-based segmentation models that may better capture irregular lesion boundaries. Moreover, it should include a formal cost-effectiveness study covering workflow integration, infrastructure requirements, reading time, recall rates, biopsy recommendations, and downstream costs. These improvements aim to maximize the clinical utility of *MassSeg-Framework* and support future evaluation in diverse healthcare environments.

## Figures and Tables

**Table 1:** Summary of detection performance at different detection thresholds for the *dINBreast* dataset.

Architecture	Threshold	mAP50 (SD)	PRE (SD)	REC (SD)	U-Test
Nano	0.4	**0.862 (0.06)**	**0.952 (0.06)**	**0.769 (0.11)**	p=1.00
	0.5	0.831 (0.07)	0.959 (0.06)	0.701 (0.13)	p=0.34
	0.6	0.809 (0.07)	0.970 (0.05)	0.652 (0.13)	p=0.12
	0.7	0.766 (0.06)	0.964 (0.06)	0.568 (0.12)	p<0.05
	0.8	0.707 (0.07)	0.967 (0.07)	0.444 (0.13)	p<0.05
	0.9	0.587 (0.05)	1.000 (0.00)	0.174 (0.10)	p<0.05
Small	0.4	0.849 (0.08)	0.980 (0.04)	0.723 (0.18)	p=0.82
	0.5	0.820 (0.07)	0.979 (0.04)	0.662 (0.16)	p=0.36
	0.6	0.760 (0.09)	0.978 (0.05)	0.541 (0.21)	p<0.05
	0.7	0.645 (0.23)	0.869 (0.30)	0.412 (0.21)	p<0.05
	0.8	0.539 (0.28)	0.800 (0.40)	0.278 (0.21)	p<0.05
	0.9	0.181 (0.28)	0.300 (0.46)	0.061 (0.10)	p<0.05
Medium	0.4	0.843 (0.07)	0.963 (0.05)	0.737 (0.13)	p=0.59
	0.5	0.816 (0.09)	0.967 (0.05)	0.681 (0.17)	p=0.20
	0.6	0.806 (0.11)	0.967 (0.06)	0.646 (0.19)	p=0.31
	0.7	0.675 (0.26)	0.886 (0.30)	0.472 (0.29)	p<0.05
	0.8	0.474 (0.33)	0.675 (0.45)	0.281 (0.25)	p<0.05
	0.9	0.140 (0.29)	0.200 (0.40)	0.080 (0.21)	p<0.05

SD: standard deviation; underlined value indicates the highest mAP50 (pivot for statistical comparison at α=0.05); bold values denote the selected best model.

**Table 2:** Summary of detection performance at different detection thresholds for the *dCBIS* dataset.

Architecture	Threshold	mAP50 (SD)	PRE (SD)	REC (SD)	U-Test
Nano	0.4	**0.709 (0.03)**	**0.778 (0.05)**	**0.601 (0.07)**	p=1.00
	0.5	0.690 (0.03)	0.825 (0.06)	0.529 (0.09)	p=0.24
	0.6	0.669 (0.04)	0.881 (0.07)	0.447 (0.11)	p<0.05
	0.7	0.612 (0.06)	0.920 (0.07)	0.301 (0.15)	p<0.05
	0.8	0.548 (0.05)	0.953 (0.05)	0.141 (0.11)	p<0.05
	0.9	0.404 (0.20)	0.775 (0.39)	0.033 (0.04)	p<0.05
Small	0.4	0.681 (0.03)	0.761 (0.04)	0.574 (0.03)	p=0.11
	0.5	0.665 (0.04)	0.799 (0.04)	0.510 (0.06)	p<0.05
	0.6	0.637 (0.06)	0.849 (0.05)	0.411 (0.11)	p<0.05
	0.7	0.589 (0.08)	0.901 (0.05)	0.275 (0.15)	p<0.05
	0.8	0.484 (0.17)	0.855 (0.29)	0.111 (0.10)	p<0.05
	0.9	0.176 (0.22)	0.342 (0.43)	0.010 (0.01)	p<0.05
Medium	0.4	0.704 (0.03)	0.786 (0.04)	0.591 (0.03)	p=0.79
	0.5	0.689 (0.03)	0.835 (0.04)	0.524 (0.07)	p=0.19
	0.6	0.640 (0.07)	0.890 (0.05)	0.381 (0.16)	p<0.05
	0.7	0.587 (0.05)	0.949 (0.06)	0.226 (0.15)	p<0.05
	0.8	0.420 (0.21)	0.763 (0.39)	0.077 (0.12)	p<0.05
	0.9	0.051 (0.15)	0.100 (0.30)	0.003 (0.08)	p<0.05

SD-standard deviation score; underlined value means the higher mAP50 score (i.e., the statistical comparison pivot at α=0.05); bold values are the best-selected model.

**Table 3:** Hardware performance metrics of detection models for the *dINBreast* dataset.

		Metric			
Architecture	Phase	Time (s)	Memory (MB)	FLOPs (G)	Params (M)
	model specs	-	-	3.307	2.624
Nano	train	275.28 (19.37)	5010.24 (17.01)	-	-
	test	280.34	4941.95	-	-
	model specs	-	-	10.859	9.459
Small	train	346.14 (45.37)	9279.78 (44.03)	-	-
	test	406.47	9272.36	-	-
	model specs	-	-	34.264	20.115
Medium	train	682.93 (95.15)	17,737.10 (7.13)	-	-
	test	794.11	17,745.92	-	-

**Table 4:** Hardware performance metrics of detection models for the *dCBIS* dataset.

		Metric			
Architecture	Phase	Time (s)	Memory (MB)	FLOPs (G)	Params (M)
	model specs	-	-	3.307	2.624
Nano	train	2177.08 (225.22)	4984.77 (9.54)	-	-
	test	3591.89	4981.39	-	-
	model specs	-	-	10.859	9.459
Small	train	2793.96 (444.17)	9157.77 (23.42)	-	-
	test	3043.72	9205.22	-	-
	model specs	-	-	34.264	20.115
Medium	train	5405.33 (565.79)	17,753.40 (42.82)	-	-
	test	6411.78	17,707.80	-	-

**Table 5:** Reported segmentation performance based on the DICE metric.

Approach	Transfer Learning	Data Augmentation	Automatic Detection	Input Dimension	CBIS	INBreast
Deep Structured Learning, Dhunge et al. [69]	no	no	no (cropped ROIs)	[40 × 40]	–	0.88
ResNet50+VGG16+Shortest Path on Graphs, Oliveira et al. [70]	yes	yes	yes	[224 × 224]	–	0.83
Multi-level nested pyramid network, Wang et al. [71]	no	no	no (cropped ROIs)	[256 × 256]	0.91	0.91
Connected-UNets, Baccouche et al. [39]	no	yes	no (cropped ROIs)	[256 × 256]	0.89	0.95
Connected-SegNets, Alkhaleefah et al. [72]	no	yes	no (cropped ROIs)	[256 × 256]	0.93	0.96
Unet128Adam, Soulami et al. [38]	no	no	yes *	[128 × 128]	–	0.99
Unet256Adam, Soulami et al. [38]	no	no	yes *	[256 × 256]	–	0.99
Associated-ResUNets, Ahmad et al. [73]	no	yes	yes *	[227 × 227]	0.95	–
Proposed *MassSeg-Framework*	no	no	yes *	[640 × 640]	0.70	0.72

* Detection in the resized image.

## Data Availability

The original DDSM (https://www.kaggle.com/datasets/cheddad/miniddsm2, accessed on 1 October 2025) and INbreast (https://www.kaggle.com/datasets/ramanathansp20/inbreast-dataset, accessed on 1 October 2025) datasets used in this study are openly available at Kaggle.
